# Assessing the Content Validity of a New Patient-Reported Measure of Barriers to Antiretroviral Therapy Adherence for Electronic Administration in Routine HIV Care: Proposal for a Web-Based Delphi Study

**DOI:** 10.2196/12836

**Published:** 2019-08-02

**Authors:** Kim Engler, Sara Ahmed, David Lessard, Serge Vicente, Bertrand Lebouché

**Affiliations:** 1 Center for Outcomes Research and Evaluation Research Institute of the McGill University Health Centre Montreal, QC Canada; 2 School of Physical & Occupational Therapy McGill University Montreal, QC Canada; 3 Centre de recherche interdisciplinaire en réadaptation (CRIR) Constance Lethbridge Rehabilitation Center Montreal, QC Canada; 4 Department of Mathematics and Statistics University of Montreal Montreal, QC Canada; 5 Department of Family Medicine McGill University Montreal, QC Canada; 6 Chronic Viral Illness Service Royal Victoria Hospital Montreal, QC Canada

**Keywords:** HIV, antiretroviral therapy, highly active, patient-reported outcome measure, medication adherence, Delphi technique, stakeholder participation, Canada, France

## Abstract

**Background:**

Adherence to lifesaving antiretroviral therapy (ART) for HIV infection remains a challenge for many patients. Routine screening for barriers to ART adherence could help make HIV care more patient-centered and prevent virologic rebound or failure. Our team is currently developing a new HIV-specific patient-reported outcome measure (PROM) of these barriers for use in Canada and France along with a digital app for its electronic administration. In our previous work, we developed the PROM’s multidimensional conceptual framework and generated 100 English items, which have been translated to French.

**Objective:**

This study aims to use a Web-based Delphi to help validate and select the content of this new HIV-specific PROM, based on the perspective of anglophone and francophone patients and providers in Canada and France. Here, we present the proposal for this Delphi.

**Methods:**

This modified Delphi will involve a diverse panel of patients (n=32) and providers (n=52) recruited especially from the 9 sites of the PROM development study (site locations in Canada: Montreal, Toronto, Vancouver; in France: Paris, Nantes, Clermont-Ferrand, Saint-Martin, Cayenne). Overall, 2 rounds of Web-based questionnaires will be conducted. The threshold for consensus is set at 60% and will determine which items are carried forward to the second round. Per item, 3 aspects will be rated: importance as a barrier to ART adherence, relevance for HIV care, and clarity. In both rounds, space will be available for free text comments. Overall comprehensiveness will be assessed in the second round.

**Results:**

This study has undergone a methodological review by experts in patient-oriented research. It has received approval from a research ethics board of the McGill University Health Centre. It is financially supported, in part, by the Canadian Institutes of Health Research’s Strategy for Patient-Oriented Research-Quebec Support Unit (M006). As of May 21, 2019, 15 people living with HIV and 25 providers completed the first round of the Delphi (24 from Canada and 16 from France).

**Conclusions:**

To our knowledge, this is the first Delphi to seek consensus on the most relevant and clinically actionable barriers to ART adherence, collecting opinions on an extensive list of barriers. Drawing on a relatively large and diverse panel of HIV patients and providers, it essentially engages key stakeholders in decision making about the PROM’s final content, helping to ensure its utility and adoption.

**International Registered Report Identifier (IRRID):**

PRR1-10.2196/12836

## Introduction

### Rationale for the New Measure

**Figure 1:**
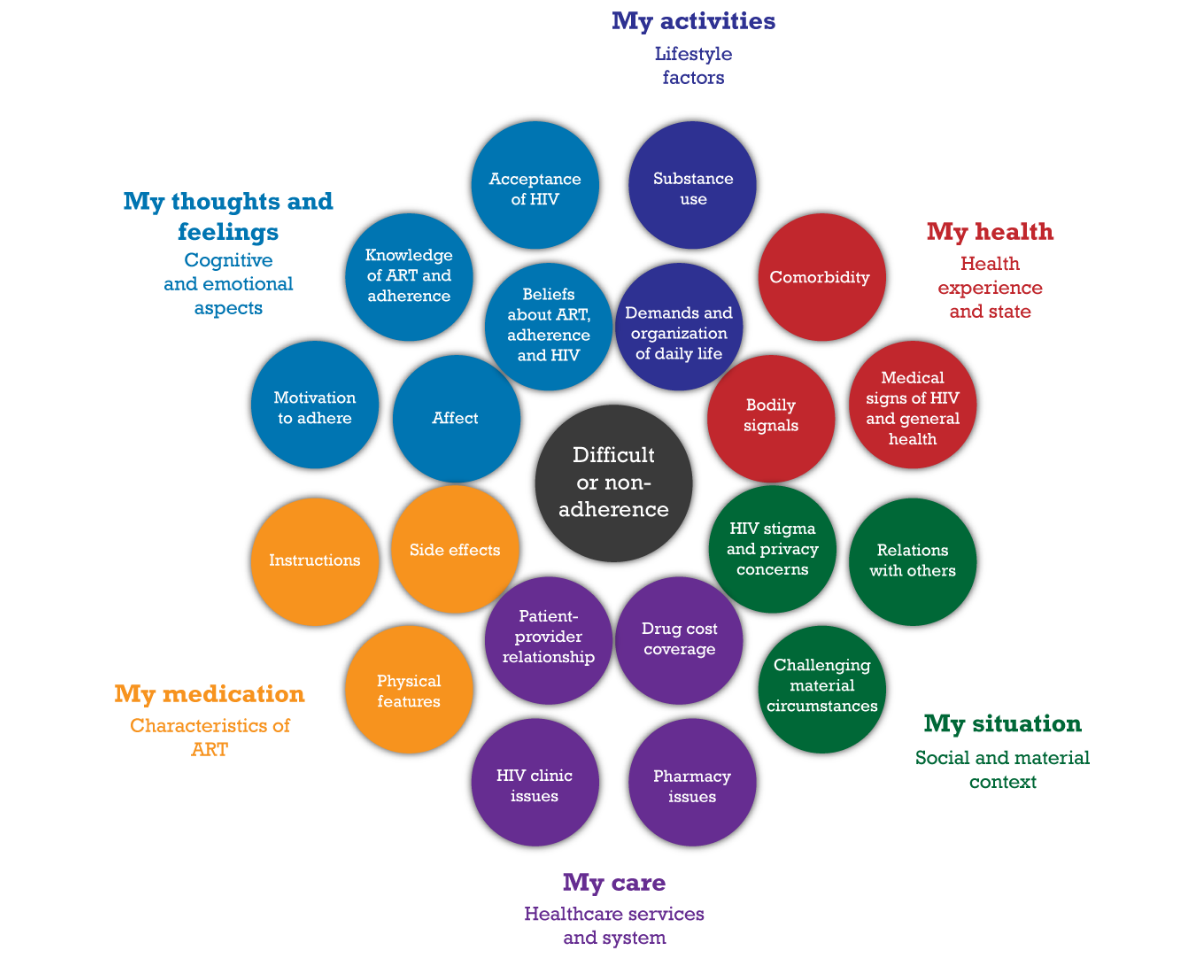
The patient-reported outcome measure’s conceptual framework of barriers to antiretroviral therapy adherence (revised from Engler et al, 2018, following the item generation and translation processes). ART: antiretroviral therapy.

The success of antiretroviral therapy (ART) for the treatment of HIV currently depends on adequate daily adherence to suppress replication of the virus. Both people living with HIV (PLHIV) and providers agree that adherence is among the top priority areas of HIV clinical care [1]. As a wide variety of factors can impede it [2], it remains a challenge for many. Approximately 40% of adult PLHIV on ART in North America and western Europe are estimated to be less than 90% adherent [3], and, thus, do not attain ideal levels of adherence. In HIV care, clinical guidelines recommend regularly identifying patients’ barriers to ART adherence [4]. However, without a tool for this purpose, a thorough in-clinic assessment may not occur. It could be hindered, for instance, by its potentially time-consuming nature [5], poor quality communication about ART adherence [6,7], or inaccurate estimation of patient adherence [8,9]. Systematically using an electronically administered patient-reported measure for this purpose could provide a relatively quick and affordable solution, offer opportunities for patient-centered counseling and intervention [5], and help prevent virologic rebound (plasma HIV ribonucleic acid (RNA) levels >200 copies/mL, following suppression of the virus) or failure (persistent HIV RNA at these levels) [4]. Yet, no extant measure of barriers to ART adherence appears to have been designed for this purpose or to be sufficiently comprehensive (Engler et al, in press). Hence, our research team is currently developing a new patient-reported outcome measure (PROM) for use in HIV care in Canada and France that will be accessible through a digital app. This electronic PROM (e-PROM) will help to routinely detect and monitor an extensive range of barriers to ART adherence. PROMs are instruments or tools that directly assess, from the patient’s perspective, their health, quality of life, or functional status associated with their health care or treatment [10]. Our preliminary work has led to the generation of a conceptual framework for the measure, which specifies multiple barrier domains for consideration and based upon which measure items were drafted. The latest version of the framework is presented in Figure 1.

### Origin of the Measure’s Conceptual Domains and Items

When developing a PROM, it is important to measure domains that are relevant to the target patients [11], in this case, PLHIV on ART. We therefore derived the domains from a synthesis of the results of 41 qualitative studies on ART adherence barriers among ART-experienced PLHIV in developed countries [12]. All included studies were published in the combination ART era, which began in 1996 (range: 1999 to 2015), with over half published from 2006 [12], when single-tablet regimens were introduced. The 6 broad domains of the conceptual framework arising from this study closely correspond with the dimensions of the World Health Organization’s model of adherence across chronic conditions [13]. The framework also has 20 distinct subdomains, which were submitted to the patient advisory committee of our PROM development study for input [12]. Relevance ratings on all subdomains indicated either unanimous relevance or top-5 priority status for 12 subdomains, with mixed ratings on the others.

From these subdomains, 100 English items were drafted, virtually all referring to a specific barrier mentioned in at least 4 (10%) of the studies included in the synthesis [12]. On the basis of recommendations for measure development to consider existing items [14], the content of well over 100 PROMs (HIV-specific and generic) of related concepts was examined for candidate items. Many examined measures can be found in our reviews of HIV-specific PROMs (Engler et al, in press; [15]). Few extant items were integrated into the draft measure verbatim. Most generated items were inspired or adapted from items within the considered measures. Items were reviewed by our research team and then checked and revised for readability with: (1) the Question Understanding Aid (University of Memphis), a free software tool available over the Web; 2) scores generated by Microsoft® Word on Flesch Reading Ease (70.1) and the Flesch-Kincaid Grade Level (6.4), indicating *fairly easy* and Grade 6 level readability, as recommended [14]; and 3) a 2-hour pilot cognitive interview with a PLHIV in Montreal.

Following guidelines for the translation and cultural adaptation of PROMs [16], we produced a French version of the questionnaire, with the help of FACIT Translation Services. In short, the English items underwent 2 forward translations by native French speakers from Canada and France, reconciliation by our research team, 2 back-translations by native English speakers fluent in French, and review and finalization by our research team. The questionnaire was also formatted and proofread by 2 translators. The proof readings were then reconciled. This process generated both English and *universal* (Canada-France) French versions of our PROM for further validation.

The French version of the PROM was not subjected to readability testing equivalent to that performed on the English version, before translation. However, readability was considered in the translation process. We also expect the Delphi and subsequent steps in our measure’s development (eg, cognitive interviews in both Canada and France) to allow further checks on the items’ readability.

### The Need for Additional Stakeholder Engagement

When considering PROM use in clinical care, it is important to consult both patients and providers [17]. In such contexts, PROM utility and adoption is associated with how *relevant, meaningful, and actionable* scores are to end users [18]. Actionability refers to the utility of the information provided for clinical decision making. Specifically, PROM scores are actionable if providers know how to translate them into concrete actions (eg, treatment adjustment and referral) [19]. Furthermore, in developing a new PROM, evidence must be generated of its content validity, which refers to the relevance, comprehensibility, and comprehensiveness of its content for a specified construct, population, and use [20]. Other standards in PROM development include consideration of user burden (eg, length) [21]. Pragmatically, in the context of busy outpatient settings, shorter PROMs may be preferable [19], for example, to limit interruptions to clinic flow. Although we have an active Montreal-based HIV patient advisory committee [22] and have conducted qualitative needs assessments with HIV clinicians in France and Canada, in the planning of our new measure and its digital app [23], further stakeholder engagement was deemed necessary to respond to the above issues.

### Study Objectives

The general objective of this study is to use a Web-based Delphi to help validate and select the content of a new HIV-specific PROM, based on the perspective of patients and providers in Canada and France. Specific objectives are to evaluate the relevance, actionability, comprehensibility, comprehensiveness, and crosscultural equivalence of the instrument’s items (eg, French-Canada vs French-France).

Here, we outline the proposal for this Delphi.

### Research Questions

The main research questions addressed and, in parentheses, the type of evidence to be examined are as follows. In the stakeholders’ experience: (1) What items reflect important barriers to ART adherence? (relevance and crosscultural equivalence), (2) What items are relevant to HIV care? that is provide useful information for medical decision making (relevance, actionability, and crosscultural equivalence), (3) Are the items clear? (comprehensibility), and (4) Do the items address all relevant barriers? (comprehensiveness).

## Methods

### The Design

The proposed study will employ Delphi survey techniques, which are consensus-building methods [24]. More specifically, a Delphi involves group facilitation to obtain opinion consensus among a panel of *experts* through several rounds of anonymously completed questionnaires [24]. The questionnaire results are summarized and returned to the participants, following each round, and structure the next round’s questionnaire [24]. This offers opportunities to panelists to change their responses, considering the group’s collective opinion. The process typically ends when consensus is achieved or returns diminish.

A Delphi is a useful and recognized method for ensuring the content validity of new measures [25], as this type of validity relies on the judgment of experts [14]. As we will especially request that participants react to previously prepared material (ie, the new measure’s items), our study design can be considered a *reactive Delphi* [26]. In avoiding a first round of open-ended questions, characteristic of a classical Delphi, it classifies as a modified Delphi [27].

### The Panel

The panel will contain 2 broad stakeholder groups, HIV patients and providers, acknowledging potential differences in opinion [28]. The perspectives of HIV patients and providers can diverge on critical aspects of HIV care [1,29], on the preferred attributes of ART [30] and on whether adherence problems are present [8]. What factors are reported to contribute to the success or failure of electronic health interventions are also found to differ between patients (eg, patient empowerment and self-management) and providers (eg, health care quality and workflow) [31]. Hence, the groups may disagree on the aspects examined.

### Sampling and Recruitment

#### Sample Size

Although variable, Delphi sample sizes of 15 to 20 participants are common [32]. With disparate or heterogeneous groups, larger samples may be required [33]. Nevertheless, in exercises involving expert content validation of a new measure, a panel of 8 to 12 experts can be considered large [34]. Given Cosmin standards for the sample sizes of quantitative content validity studies [20] and our budgetary limitations (see section on compensation), we decided to recruit 52 HIV health care, social and community service providers (ie, 12 each of physicians, nurses, and pharmacists; 5 each of social workers and psychologists or psychiatrists; 6 staff members of community-based organizations (CBOs); and 32 ART-experienced HIV patients. For Cosmin, 30 to 49 participants per group is deemed *adequate*, whereas 50 or greater is considered *very good*, the highest possible rating [20]. Half of the panel will be recruited from each country (Canada and France).

#### Inclusion Criteria

A Delphi is usually conducted with panelists who possess subject matter expertise on the given topic [25]. Sampling will thus be purposeful [35]. *Expertise* among HIV providers is arbitrarily defined as having at least 5 years of clinical practice experience with HIV patients. Among HIV patients, *expertise* is required in taking ART to control HIV infection and facing barriers to its use. HIV-positive persons will be eligible if they are aged at least 18 years, have been prescribed ART for at least 1 year, irrespective of current use of ART, and if, based on self-report, in the past 5 years, they had difficulty adhering to ART, as prescribed. This will help ensure that participants are knowledgeable of barriers to adhering to *current* ART regimens. Participants will need to confirm their easy access to the internet and comfort completing Web-based questionnaires in either English or French.

#### Recruitment

HIV providers will mainly be recruited within the 9 participating sites of the PROM development study in Canada and France, which is described elsewhere [22]. Patients will mainly be recruited by referral from providers at the participating clinics or through community organizations within the clinics’ cities (in Canada: Montreal, Toronto, and Vancouver; in France: Paris, Nantes, Clermont-Ferrand, Saint-Martin, and Cayenne). Patient participants in Canada will be administratively included through the main study site, the Chronic Viral Illness Service of the McGill University Health Centre in Montreal, Quebec. At least 25% of participants recruited for each panel will be cisgender women, to ensure their equitable inclusion [36]. This lower limit is close to the estimated average proportion of PLHIV who are women in Canada (23% [37]) and France (32% [38]). At least 50% of providers and patients recruited in Canada will be fluent in English to allow adequate evaluation of each language version of the measure. A range of HIV providers that intervene on matters of adherence will be sought. Per country, we will recruit 6 clinicians, 6 pharmacists, 6 nurses, 2 to 3 social workers, 2 to 3 psychologists or psychiatrists, and 3 staff members of CBOs. Representation of at least 3 Canadian cities and 3 cities or collectivities in France will be sought for both patients and providers.

Gatekeepers will help identify eligible HIV providers [24] (eg, directors of the participating sites’ HIV services and actors within the pharmaceutical industry). Emails will be sent to candidates within each site inviting them to participate in the Delphi. Those who accept will be directed to a secure website where they will be guided through the consent process [39].

In the participating sites, HIV patients will be approached by health care providers or research staff and referred to a designated staff member, if interested in the study. Eligibility and adequate inclusion of women will be verified among those who wish to participate. As with providers, eligible individuals, who accept, will be directed to a secure website where they will be guided through the consent process [39].

### Delphi Procedure

The planned Delphi structure will involve only 2 rounds of data collection with Web-based questionnaires to limit costs, respondent burden, and attrition. To develop all participant questionnaires and acquire informed consent, we will use the SurveyMonkey software (SurveyMonkey Inc, San Mateo, California, USA). Before its use, the round 1 questionnaire, available in French or English, will be piloted with at least 2 clinicians and 2 patients. Once an individual has accepted to participate, has consented, and completed a brief survey on their characteristics, they may begin the Delphi’s first round, which involves completing the associated questionnaire within 2 weeks. Within 2 weeks of receiving the panel’s full data, feedback will be given to the panelists in the form of a report, detailing areas of consensus and disagreement. Any specific instructions for round 2 will be sent with the round 1 report, requesting panelist responses, again, within 2 weeks. Within 2 weeks of receiving round 2’s data, a second report will be provided to panelists, describing the final results.

#### Reminders

Although ultimately under participant control, maintaining involvement is important to a Delphi’s rigor and reminders can be used to enhance response rates [24]. A reminder will be sent to participants 1 week before the official deadline for completing the Web-based questionnaires. If necessary, up to 2 additional weekly reminders will be sent, if no data are received. Participants will be *asked* to complete the questionnaire within 2 weeks, but we will accord them a maximum of 4 weeks before considering them lost to follow up.

#### Measures to Ensure Anonymity

We will ensure the anonymity of panelists to each other but not to members of the research team. This *quasianonymity* is necessary to ensure follow-up of nonresponding participants [24] and to provide compensation for completed rounds. Standard measures to ensure research participant confidentiality will be in place.

#### Compensation

Attrition is particularly concerning in Delphi studies, because of their multiple rounds [27]. Delphi studies with HIV providers can document decreases in response rates from the first to the second round, from 33% to 0% (33% [1]; 20% [40]; 0% [39]). A decrease of 46% was reported in a recent Delphi study with HIV patients [41]. To foster retention, participants will be compensated for each completed Delphi round upon receipt and verification of their Web-based questionnaire data. Compensations set correspond to acceptable levels, as judged by the research ethics board (REB) that evaluated the study. PLHIV and community organization staff will be compensated the equivalent of $50 Canadian per round. Health and social service professionals will receive the equivalent of $100 Canadian per round. The differential is partially explained by ethical concerns about undue inducement of patients and the felt need to sufficiently incentivize professionals to ensure their participation. At consent, participants will also indicate if they wish to be acknowledged in any study or presentation arising from the Delphi results, provided they complete both rounds.

### Instructions

Following the Web-based consent process, both patients and providers will complete a brief survey to allow description of the panel. Both groups will provide information on their sociodemographic characteristics, whereas providers will answer additional questions on their HIV clinical practice and patients, on their HIV treatment (eg, whether currently on ART). For both Delphi rounds, patients and providers will complete identical Web-based questionnaires and receive instructions in simple language.

#### Round 1

Round 1 will be the most intensive for participants. The respondents’ tasks will be, for each item proposed, to rate its (1) importance as a barrier to ART adherence (ie, Is this an important barrier to properly taking ART?), (2) relevance for HIV care (ie, Is this useful information for HIV care?), and (3) clarity (ie, Is the item clearly written? Does it make sense?). They will also provide comments, as needed (eg, suggested corrections and new items). For the list of items evaluated, contact the corresponding author. 

#### Round 2

After considering the feedback provided in the round 1 report, respondents’ main tasks for round 2 will be to review and rate the contested items on 1 to all of the same 3 aspects: (1) importance as a barrier, (2) relevance for HIV care, and (3) clarity (following item modification, if applicable). The measure’s overall comprehensiveness will also be rated as will be any respondent-proposed items, if included.

### Response Options

Relevance and importance will be measured with a slightly adapted frequently used four-point ordinal scale, appropriate for this purpose [34]: (1) No, (2) Somewhat, (3) Quite, and (4) Very. For simplicity, the same response scale will be used for item clarity and measure comprehensiveness. For each item, space for free text comments will be provided. Table 1 illustrates the question and response structure for each item evaluated at round 1. These will be modified for round 2, including only scales for an item’s contested aspect(s).

**Table 1:** Responses collected for each patient-reported outcome measure item during round 1 of the Delphi.

Item		Answers			
Is this item...					
	an important barrier?	No	Somewhat	Quite	Very
	relevant for HIV care?	No	Somewhat	Quite	Very
	clear?	No	Somewhat	Quite	Very
Comments (optional):					

### Analyses

#### Determining Consensus

In the absence of standards for determining consensus in Delphi studies [43], our criterion was chosen to foster inclusivity and recognition of each stakeholder group’s respective interests. As the measure contains items that are *causal indicators*, that is, they define the construct (ART adherence barriers) rather than being defined by it [14], inclusivity was doubly important. Exclusion of such items could lead to the underestimation of ART adherence barriers [14]. However, a 100-item measure seems impractical for routine use in HIV care.

For these reasons, we determined *a priori* that *consensus* on an item would be achieved if *60% or more* of either group (ie, 60% among patients or providers) or within the panel (ie, 60% of all participants) agree (ie, on importance, relevance, or clarity). In total, 60% is within the range of suggested proportions for determining consensus [26].

For example, if at least 60% of one group or of all participants agree that an item is important (ie, a score of 3 or 4 on the 4-point rating scale), consensus will be considered reached, as for relevance. For clarity, if minimum 60% of at least one stakeholder group or of all participants agree an item is not or only *somewhat* clear (ie, a score of 1 or 2), it will be reformulated and carried forward into round 2.

Items demonstrating consensus on importance and relevance, without clarity problems (as defined above), will not be carried forward into round 2. Any remaining items not meeting this condition after round 2 will be reevaluated. Specifically, given the complexity of the results generated by this Delphi and the panel’s diversity, a multidisciplinary committee formed of investigators, providers, patients, psychometricians, and other experts will be constituted to review the final Delphi results and many potentially relevant comparisons (eg, by country and sex). The committee will make decisions about item removal or inclusion for the PROM and about which results to prioritize in the process.

#### Evaluating Content Validity

Consensus, as defined earlier, will determine the inclusion or exclusion of questionnaire items in round 2. However, for informative purposes, the content validity of each item will be calculated with the item-content validity index (I-CVI) [34]. This index will represent the proportion of experts in agreement about relevance and importance (ie, the number of experts scoring 3 or 4, divided by the total number of experts). As suggested by Polit et al [34], items with an I-CVI score of .78 or above will be considered to have an excellent content validity. This assessment of content validity will take account of I-CVI score comparisons across groups (eg, patients vs providers) and be considered by the multidisciplinary committee.

#### Exploring Crosscultural Equivalence

Crosscultural equivalence will be examined based on *item equivalence*, which concerns the relevance of items to the target population [14]. We will, therefore, compare the final I-CVI item relevance and importance scores of anglophones with those of francophones and compare the scores of both francophone respondent groups (Canada vs France). This exercise will be exploratory; equivalence (eg, conceptual and measurement) will be further investigated in subsequent steps of the PROM development study (eg, with cognitive interviews and psychometric validation).

#### Descriptive Statistics

At each round, proportions endorsing each response option and measures of central tendency will be calculated (ie, medians and modes) [42], for all aspects considered.

#### Comparative Analyses

For descriptive purposes, statistical analyses of group comparisons (eg, patients vs providers) with nonparametric tests, appropriate with ordinal data (eg, Chi square and Fisher exact test with dichotomized variables) [43], will be conducted and shared with the participants. Tests of the stability of opinions on contested items from rounds 1 to 2 will also be performed (eg, with Wilcoxon matched-pairs signed-ranks test) [43].

#### Qualitative Analysis

If respondents provide sufficient written comments, they will be submitted to content analysis [44] to inform the interpretation of results.

#### Adding New Items

If new items are suggested at round 1 in the comments, they will be compared with the existing items and discussed by the research team to decide if they should be evaluated by panelists in round 2.

### Feedback at Each Round

In line with recommendations and to stimulate consensus [42] and motivation, after a round, each participant will be given a report showing how their scores relate to the global results. Results will be presented for the full panel and stratified by general stakeholder group (patients, providers). A selection of all analyses conducted will be included in the report, to not overburden participants with information.

## Results

### Scientific and Ethics Reviews

This Delphi received methodological review by the Canadian Institutes of Health Research (CIHR), Strategy for Patient-Oriented Research (SPOR)-Quebec Support Unit, between March and May 2018, from Delphi experts on the Method Development team. The McGill University Health Center REB evaluated it as an amendment to the previously approved e-PROM development study. It granted approval on November 30, 2018.

### Stakeholder Feedback on the Study and Contributions to Date

In November 2018, the Delphi Study was presented for feedback to our Montreal-based Knowledge Users Committee, which includes a strong representation of CBOs. As a result, it was decided to include CBO staff on the expert panel. In December 2018, the round 1 questionnaire was piloted in Montreal with 2 providers (a francophone pharmacist and an anglophone nurse) and 2 female patients (1 francophone and 1 anglophone), leading to more specific instructions to patients. To help limit the impact of computer literacy and internet access on patient participation, in partnership with a CBO, AIDS Community Care Montreal, a computer workshop and terminals were available to potential participants, leading to the inclusion of 5 participants in April 2018. There are also plans through our partnership with this CBO to include an incarcerated individual who would complete the questionnaire on paper.

### Recruitment and Participants to Date

On May 21, 2019, of 58 individuals who were sent the invitation and survey link, 47 opened the invitation, 10 did not, and 1 opted out. Among these, 40 (69%) provided complete data, which represents 48% of our recruitment goal (40/84). The median time to complete the round 1 questionnaire was 1 h 55 min.

Respondents with complete data included 15 PLHIV and 25 providers, with 24 individuals from Canada and 16 from France. Among providers, there were 18 women and 7 men. Those in Canada were from Montreal (n=11), Toronto (n=4), and Vancouver (n=1). Those in France were from Paris (n=8) and Clermont Ferrand (n=1). The provider categories represented were pharmacist (n=8), nurse (n=7), physician (n=6), psychiatrist or psychologist (n=2), social worker (n=1), and CBO staff (n=1). Their year of birth ranged from 1955 to 1988. Over half had 15 or more years of experience treating PLHIV (13/25) and worked exclusively in a hospital setting (14/25). Among PLHIV, there were 7 women, 7 men, and 1 transgender person. They were from Montreal, Canada (n=9), and Clermont Ferrand (n=4) and Paris, France (n=2). Their year of birth ranged from 1947 to 1999. Approximately half had immigrated to their country of residence (7/15) and described their sexual orientation as heterosexual (8/15). Over a quarter (4/15) had ever injected drugs. All were currently on ART, with a quarter (3/14) reporting not being satisfied with their latest ART regimen.

### Study Duration and Deliverables

From the scale up of recruitment (April 2019), the Delphi is expected to be led over 6 months. Study findings will be communicated through peer-reviewed publications, conference presentations, and other forms of knowledge dissemination (eg, academic rounds and Web-based reports in partnership with CBOs).

## Discussion

To our knowledge, this is the first Delphi to seek consensus on the most important and clinically actionable barriers to ART adherence, drawing on a relatively large and diverse panel of HIV patients and providers. For the e-PROM’s intended use in routine HIV care in Canada and France, the Delphi will serve to identify items that should be accorded priority or that require revision for clarity. Country and language group differences in ratings will also provide indications of the crosscultural validity of the measure items. Essentially, this Delphi will engage important stakeholders in decision making about the measure’s final content, helping to ensure its utility and adoption.

Routine e-PROM collection in HIV health service provision is in its infancy, despite notable initiatives [8,45,46]. The clinical use of PROMs could have cascading effects on the delivery and outcomes of care, including potentially improving patient-provider communication, self-management, and adherence [47]. Importantly, for HIV treatment, it could help it achieve its goals not only of viral suppression, but also of quality of life [4]. Evaluating these potentialities is a part of our e-PROM research program.

## Appendix

Multimedia Appendix 1 Untitled.

## Abbreviations

ART: antiretroviral therapy
CBO: community-based organization
CIHR: Canadian Institutes of Health Research
e-PROM: electronic PROM
I-CVI: item-content validity index
PLHIV: people living with HIV
PROM: patient-reported outcome measure
REB: research ethics board
SPOR: Strategy for Patient-Oriented Research

## References

[1] Fredericksen RJ, Edwards TC, Merlin JS, Gibbons LE, Rao D, Batey DS, Dant L, Páez E, Church A, Crane PK, Crane HM, Patrick DL (2015). Patient and provider priorities for self-reported domains of HIV clinical care. AIDS Care.

[2] Shubber Z, Mills EJ, Nachega JB, Vreeman R, Freitas M, Bock P, Nsanzimana S, Penazzato M, Appolo T, Doherty M, Ford N (2016). Patient-reported barriers to adherence to antiretroviral therapy: a systematic review and meta-analysis. PLoS Med.

[3] Ortego C, Huedo-Medina TB, Llorca J, Sevilla L, Santos P, Rodríguez E, Warren MR, Vejo J (2011). Adherence to highly active antiretroviral therapy (HAART): a meta-analysis. AIDS Behav.

[4] (2018). AIDS Info.

[5] Genberg BL, Lee Y, Rogers WH, Wilson IB (2015). Four types of barriers to adherence of antiretroviral therapy are associated with decreased adherence over time. AIDS Behav.

[6] Barfod TS, Hecht FM, Rubow C, Gerstoft J (2006). Physicians' communication with patients about adherence to HIV medication in San Francisco and Copenhagen: a qualitative study using grounded theory. BMC Health Serv Res.

[7] Laws MB, Beach MC, Lee Y, Rogers WH, Saha S, Korthuis PT, Sharp V, Wilson IB (2013). Provider-patient adherence dialogue in HIV care: results of a multisite study. AIDS Behav.

[8] Fredericksen R, Crane PK, Tufano J, Ralston J, Schmidt S, Brown T, Layman D, Harrington RD, Dhanireddy S, Stone T, Lober W, Kitahata MM, Crane HM (2012). Integrating a web-based, patient-administered assessment into primary care for HIV-infected adults. J AIDS HIV Res.

[9] Phillips LA, Leventhal EA, Leventhal H (2011). Factors associated with the accuracy of physicians' predictions of patient adherence. Patient Educ Couns.

[10] Weldring T, Smith SM (2013). Patient-reported outcomes (PROs) and patient-reported outcome measures (PROMs). Health Serv Insights.

[11] Francis DO, McPheeters ML, Noud M, Penson DF, Feurer ID (2016). Checklist to operationalize measurement characteristics of patient-reported outcome measures. Syst Rev.

[12] Engler K, Lènàrt A, Lessard D, Toupin I, Lebouché B (2018). Barriers to antiretroviral therapy adherence in developed countries: a qualitative synthesis to develop a conceptual framework for a new patient-reported outcome measure. AIDS Care.

[13] (2003). World Health Organization.

[14] Streiner DL, Norman GR, Cairney J (2015). Health Measurement Scales: A Practical Guide to Their Development and Use, 5th Edition.

[15] Engler K, Lessard D, Lebouché B (2017). A review of HIV-specific patient-reported outcome measures. Patient.

[16] Wild D, Grove A, Martin M, Eremenco S, McElroy S, Verjee-Lorenz A, Erikson P, ISPOR Task Force for Translation and Cultural Adaptation (2005). Principles of good practice for the translation and cultural adaptation process for patient-reported outcomes (PRO) measures: report of the ISPOR task force for translation and cultural adaptation. Value Health.

[17] Kwan BM, Sills MR, Graham D, Hamer MK, Fairclough DL, Hammermeister KE, Kaiser A, de Jesus DP, Schilling LM (2016). Stakeholder engagement in a patient-reported outcomes (PRO) measure implementation: a report from the SAFTINet practice-based research network (PBRN). J Am Board Fam Med.

[18] Ahmed S, Ware P, Gardner W, Witter J, Bingham CO, Kairy D, Bartlett SJ (2017). Montreal accord on patient-reported outcomes (PROs) use series - paper 8: patient-reported outcomes in electronic health records can inform clinical and policy decisions. J Clin Epidemiol.

[19] Kroenke K, Monahan PO, Kean J (2015). Pragmatic characteristics of patient-reported outcome measures are important for use in clinical practice. J Clin Epidemiol.

[20] Terwee CB, Prinsen CA, Chiarotto A, Westerman MJ, Patrick DL, Alonso J, Bouter LM, de Vet HC, Mokkink LB (2018). COSMIN methodology for evaluating the content validity of patient-reported outcome measures: a Delphi study. Qual Life Res.

[21] Reeve BB, Wyrwich KW, Wu AW, Velikova G, Terwee CB, Snyder CF, Schwartz C, Revicki DA, Moinpour CM, McLeod LD, Lyons JC, Lenderking WR, Hinds PS, Hays RD, Greenhalgh J, Gershon R, Feeny D, Fayers PM, Cella D, Brundage M, Ahmed S, Aaronson NK, Butt Z (2013). ISOQOL recommends minimum standards for patient-reported outcome measures used in patient-centered outcomes and comparative effectiveness research. Qual Life Res.

[22] Engler K, Lessard D, Toupin I, Lènàrt A, Lebouché B (2017). Engaging stakeholders into an electronic patient-reported outcome development study: on making an HIV-specific e-PRO patient-centered. Health Policy Technol.

[23] Toupin I, Engler K, Lessard D, Wong L, Lènàrt A, Spire B, Raffi F, Lebouché B (2018). Developing a patient-reported outcome measure for HIV care on perceived barriers to antiretroviral adherence: assessing the needs of HIV clinicians through qualitative analysis. Qual Life Res.

[24] Hasson F, Keeney S, McKenna H (2000). Research guidelines for the Delphi survey technique. J Adv Nurs.

[25] Hatcher T, Colton S (2007). Using the internet to improve HRD research: the case of the web-based Delphi research technique to achieve content validity of an HRD-oriented measurement. J Eur Ind Train.

[26] McKenna HP (1994). The Delphi technique: a worthwhile research approach for nursing?. J Adv Nurs.

[27] Keeney S, McKenna H, Hasson F (2011). The Delphi Technique in Nursing and Health Research.

[28] Sinha IP, Smyth RL, Williamson PR (2011). Using the Delphi technique to determine which outcomes to measure in clinical trials: recommendations for the future based on a systematic review of existing studies. PLoS Med.

[29] Mosack KE, Wandrey RL (2015). Discordance in HIV-positive patient and health care provider perspectives on death, dying, and end-of-life care. Am J Hosp Palliat Care.

[30] Yelverton V, Ostermann J, Hobbie A, Madut D, Thielman N (2018). A mixed methods approach to understanding antiretroviral treatment preferences: what do patients really want?. AIDS Patient Care STDS.

[31] Granja C, Janssen W, Johansen MA (2018). Factors determining the success and failure of eHealth interventions: systematic review of the literature. J Med Internet Res.

[32] Hsu CC, Sandford BA (2007). Practical Assessment, Research & Evaluation.

[33] Skulmoski GJ, Hartman FT, Krahn J (2007). The Delphi method for graduate research. J Info Technol Educ.

[34] Polit DF, Beck CT, Owen SV (2007). Is the CVI an acceptable indicator of content validity? Appraisal and recommendations. Res Nurs Health.

[35] Etikan I, Musa SA, Alkassim RS (2016). Comparison of convenience sampling and purposive sampling. Am J Theor Appl Stat.

[36] Loutfy MR, Kennedy L, Mohammed S, Wu W, Muchenje M, Masinde K, Salam K, Soje L, Gregorovich S, Tharao W (2014). Recruitment of HIV-positive women in research: discussing barriers, facilitators, and research personnel's knowledge. Open AIDS J.

[37] Public Health Agency of Canada Government of Canada.

[38] (2017). UNAIDS.

[39] Johnson MO, Koester KA, Wood T, Neilands TB, Pomeranz JL, Christopoulos KA (2017). Development of an index of engagement in HIV care: an adapted internet-based Delphi process. JMIR Res Protoc.

[40] Sowell RL (2000). Identifying HIV/AIDS research priorities for the next millennium: a Delphi study with nurses in AIDS care. J Assoc Nurses AIDS Care.

[41] Ledgister K, Fleming-Castaldy RP (2017). The perceptions of persons living with human immunodeficiency virus/acquired immune deficiency syndrome about their experiences in regaining productive occupations: a Delphi study. Occup Ther Ment Health.

[42] Powell C (2003). The Delphi technique: myths and realities. J Adv Nurs.

[43] von der Gracht HA (2012). Consensus measurement in Delphi studies: review and implications for future quality assurance. Technol Forecast Soc Change.

[44] Elo S, Kyngäs H (2008). The qualitative content analysis process. J Adv Nurs.

[45] Kozak MS, Mugavero MJ, Ye J, Aban I, Lawrence ST, Nevin CR, Raper JL, McCullumsmith C, Schumacher JE, Crane HM, Kitahata MM, Saag MS, Willig JH (2012). Patient reported outcomes in routine care: advancing data capture for HIV cohort research. Clin Infect Dis.

[46] Barger D, Leleux O, Conte V, Sapparrart V, Gapillout M, Crespel I, Erramouspe M, Delveaux S, Dabis F, Bonnet F (2018). Integrating electronic patient-reported outcome measures into routine HIV care and the ANRS CO3 aquitaine cohort's data capture and visualization system (QuAliV): protocol for a formative research study. JMIR Res Protoc.

[47] Santana MJ, Feeny D (2014). Framework to assess the effects of using patient-reported outcome measures in chronic care management. Qual Life Res.

