# Supplementary figures and images for: Assessing the Content Validity of a New Patient-Reported Measure of Barriers to Antiretroviral Therapy Adherence for Electronic Administration in Routine HIV Care: Proposal for a Web-Based Delphi Study
Source: JMIR Res Protoc. 2019 Aug 2;8(8):e12836. doi: 10.2196/12836 (PMC6696859; doi:10.2196/12836)

## Slide 1
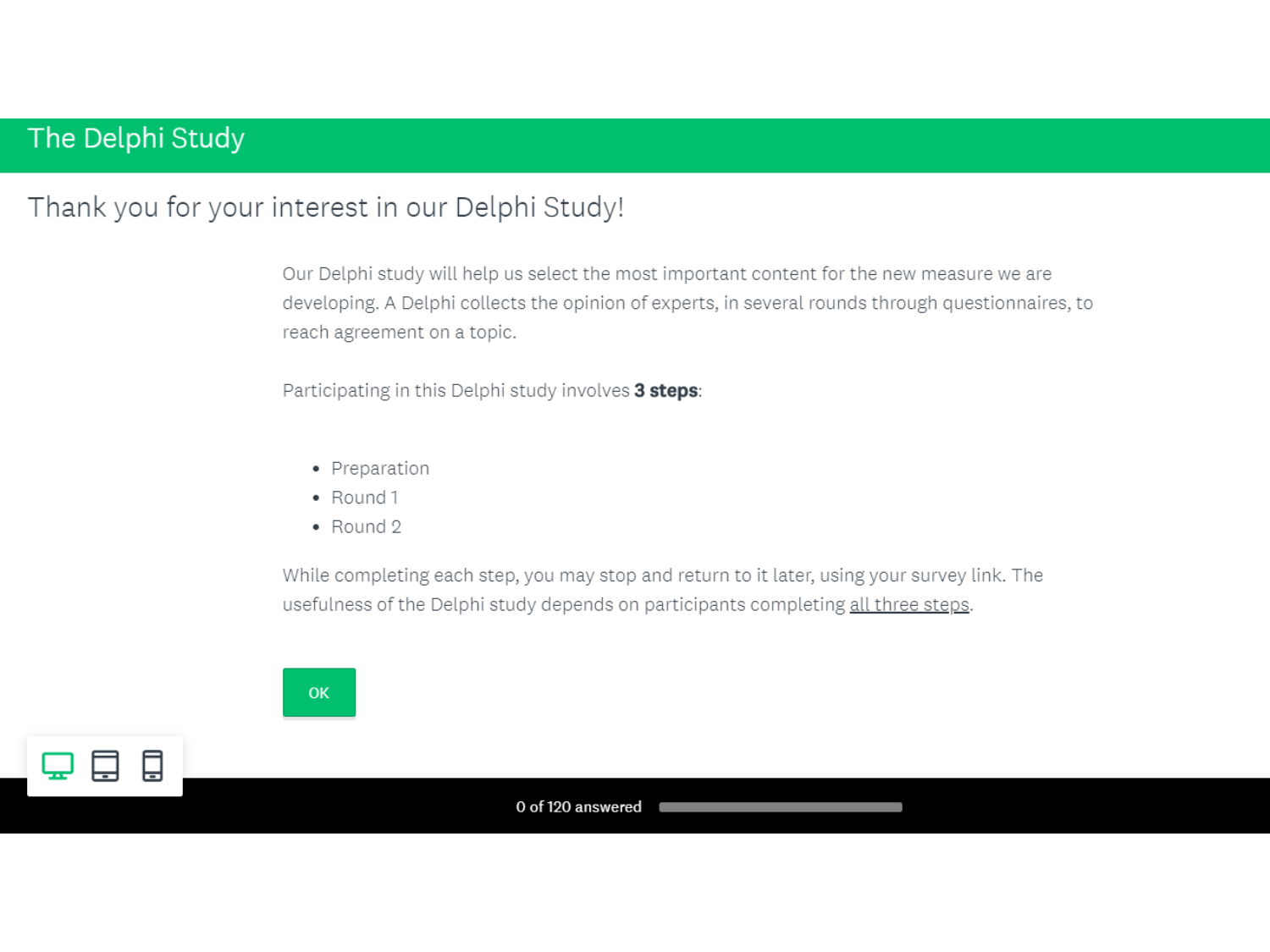

## Slide 2
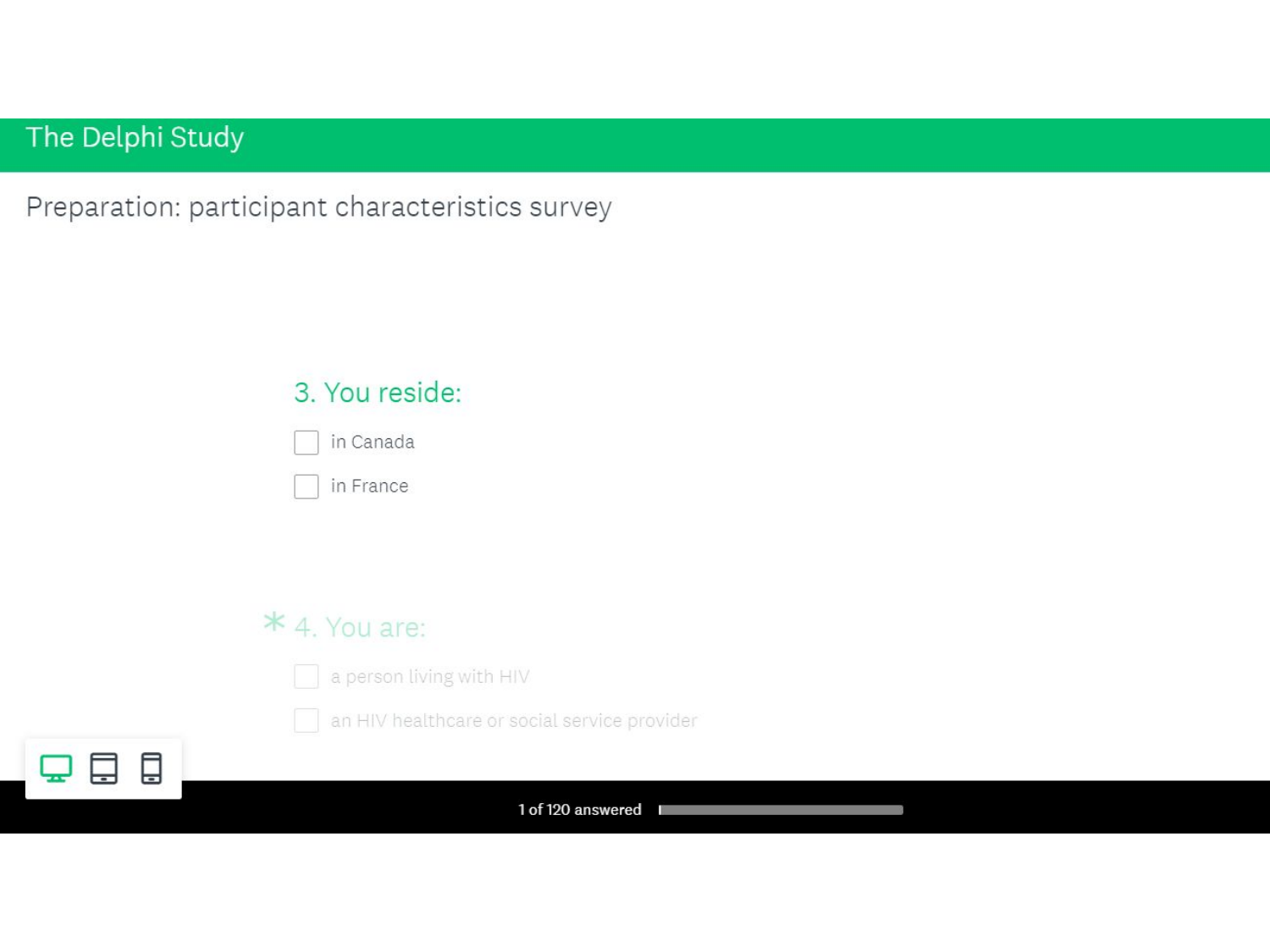

## Slide 3
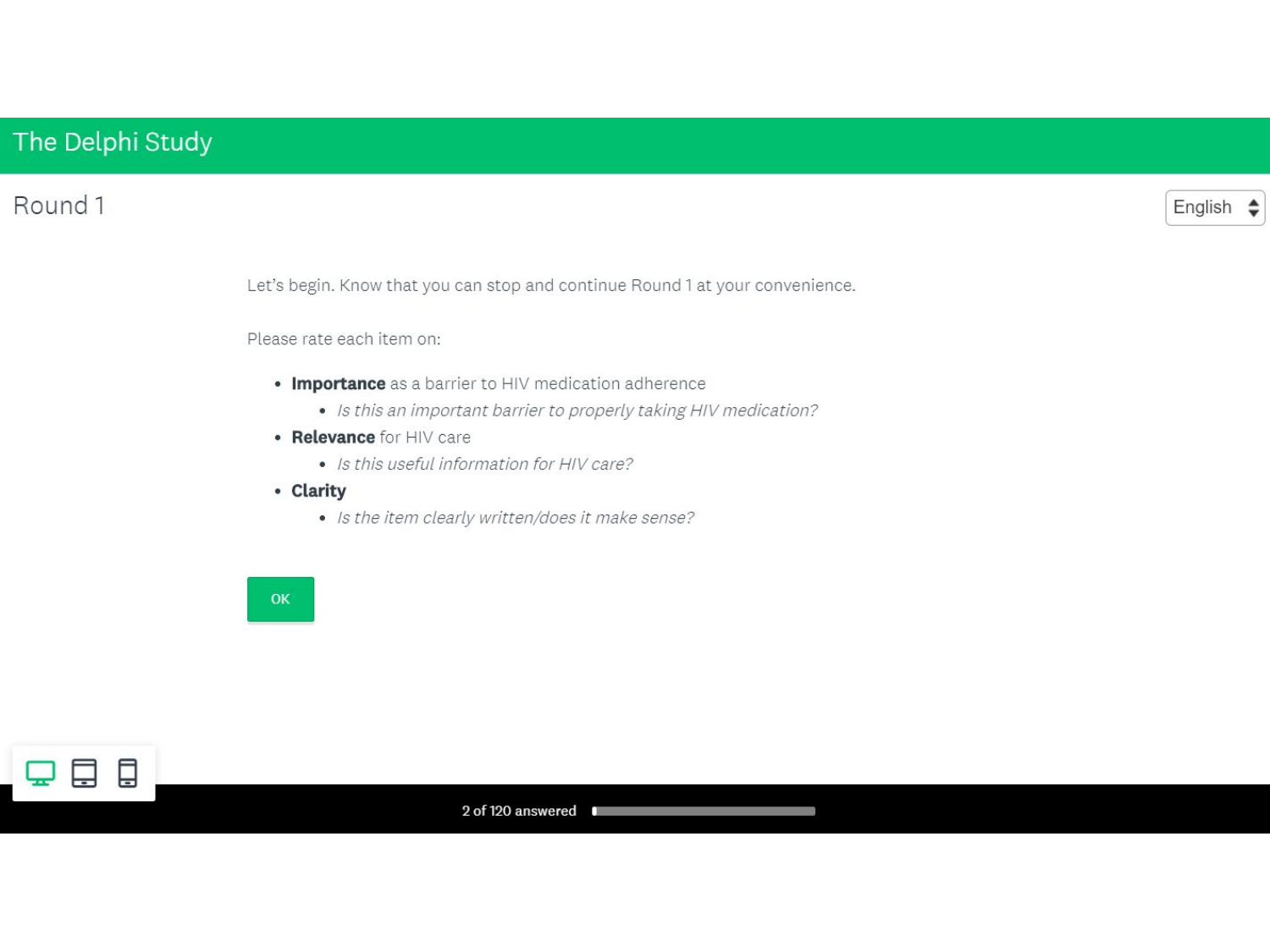

## Slide 4
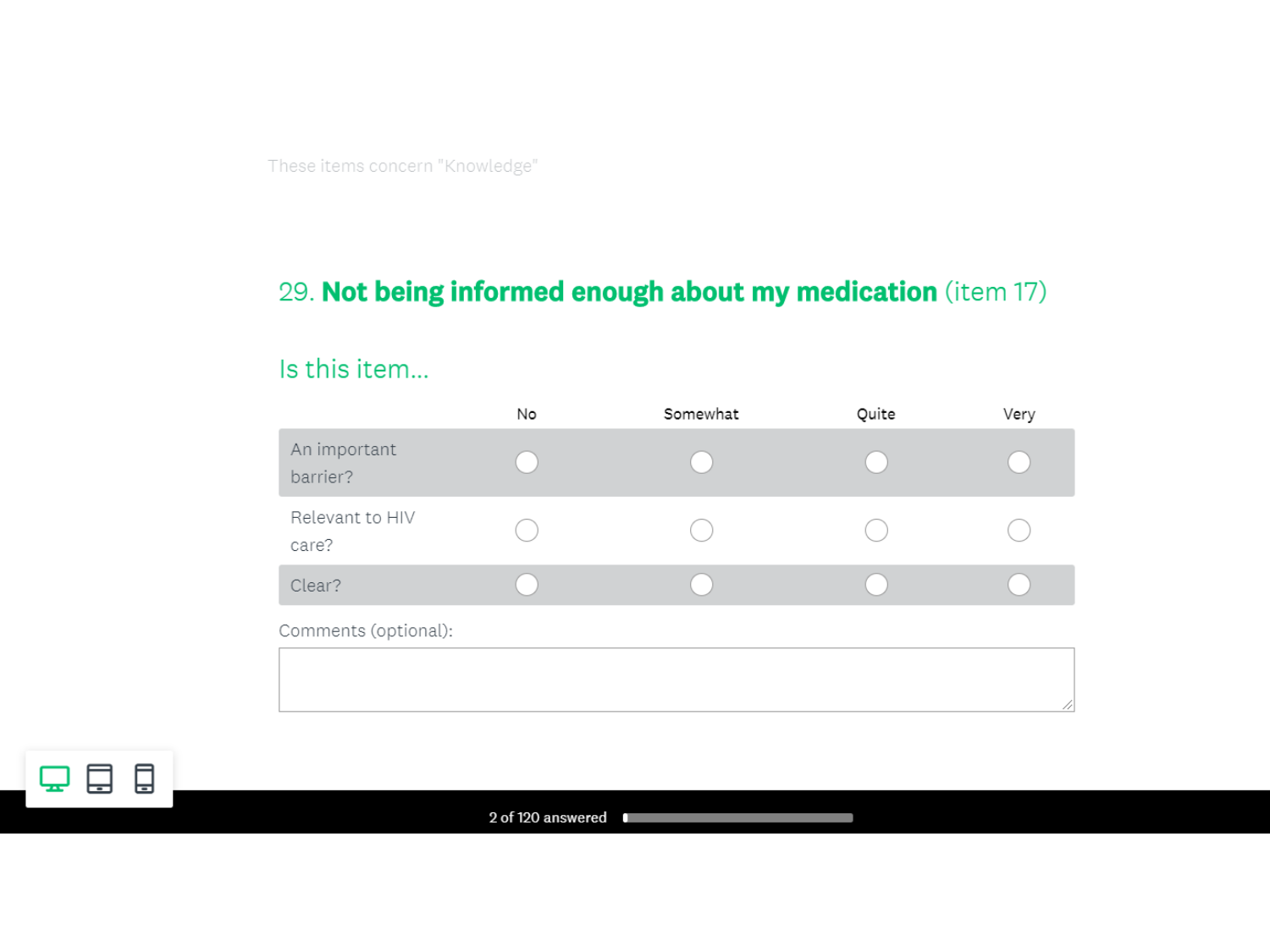

## Slide 5
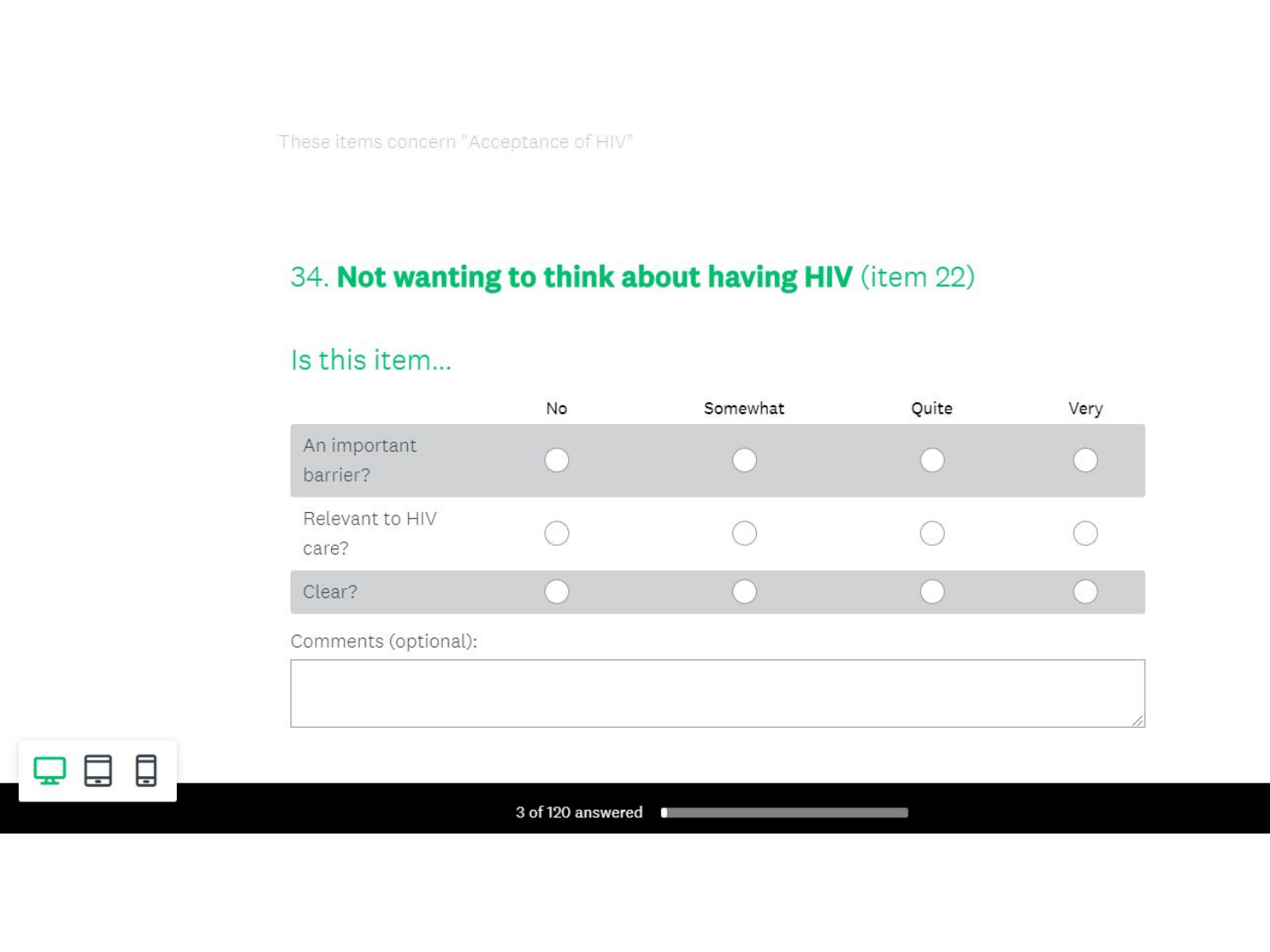

## Slide 6
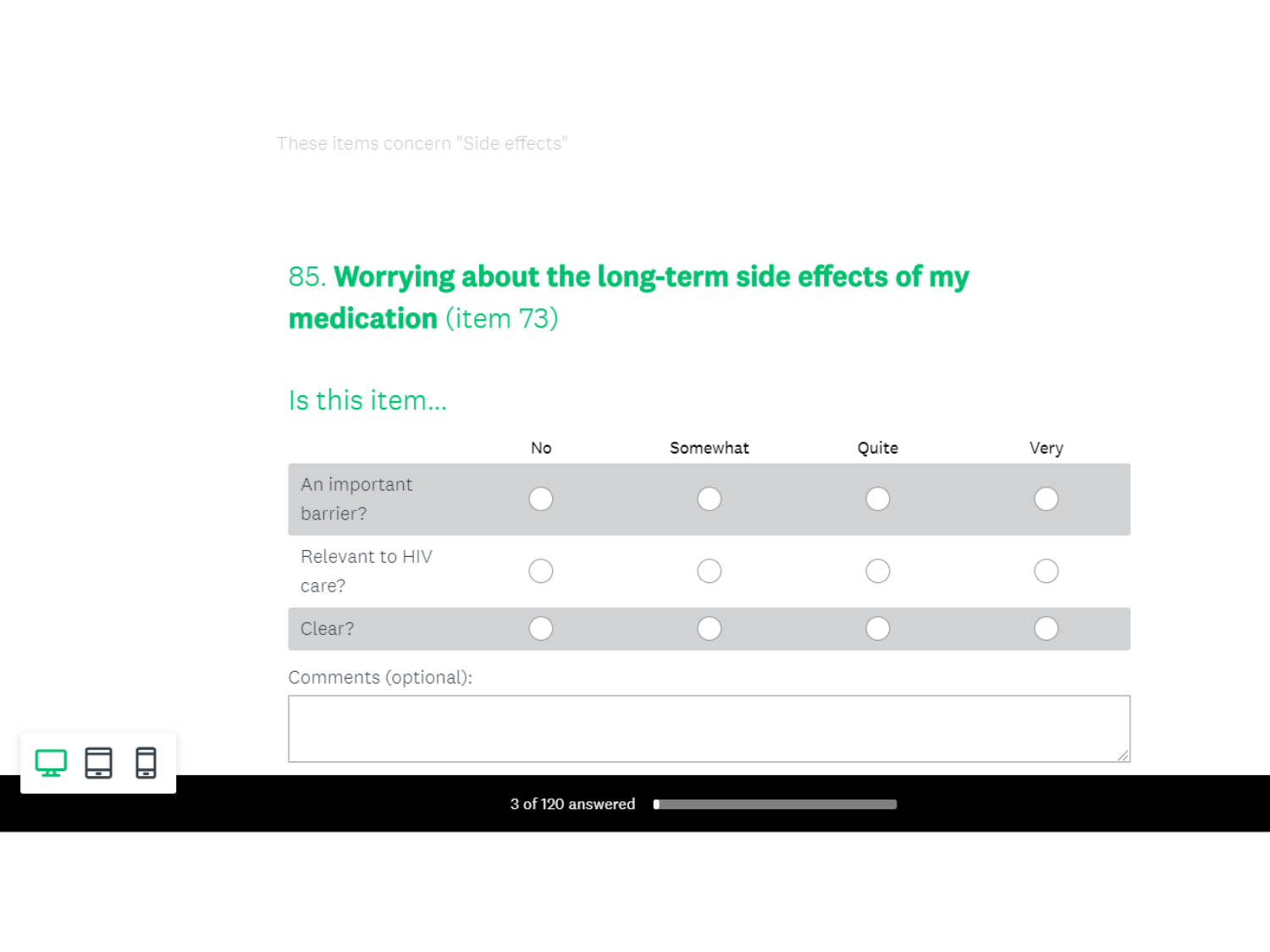

Supplement: Multimedia Appendix 1 [file resprot_v8i8e12836_app1.pptx]
